# Factors Associated With Biosecurity Preparedness for Foreign Animal Diseases Among Swine Farms in the United States Territory of Puerto Rico

**DOI:** 10.1155/tbed/3769536

**Published:** 2026-09-14

**Authors:** Jessica P. Mateus, Kara Flaherty, Isha Agrawal, Andreia G. Arruda

**Affiliations:** ^1^ Department of Veterinary Preventive Medicine, College of Veterinary Medicine, The Ohio State University, Columbus, Ohio, USA, osu.edu

**Keywords:** farm biosecurity, foreign animal disease, multispecies, small-scale pig production

## Abstract

Successful responses to foreign animal diseases (FADs) such as African Swine Fever (ASF) in swine populations depend on the timely planning and implementation of effective biosecurity measures at the farm level. This study aimed to characterize biosecurity preparedness among swine farms in Puerto Rico and to identify geographic areas with higher biosecurity‐related vulnerabilities across the island. Data were collected from 95 swine farms from seven regions using an adapted form of the enhanced biosecurity plan templates available from the Secure Pork Supply (SPS) Plan, a nationally recognized framework. Biosecurity scores were calculated using 37 captured variables related to biosecurity measures and transformed into a percentage. The overall average biosecurity score was 42.8% ± 4.5%. Lower regional‐level biosecurity scores were observed in western regions. Although some operational practices, such as feed storage management and the use of dedicated vehicles and equipment, showed higher levels of adoption, critical components such as the availability of equipment necessary for depopulation and manure storage capacity to prevent the introduction and spread of highly relevant pathogens were often lacking, particularly regarding producer‐level knowledge and preparedness. Overall, farms housing multiple species showed significantly lower biosecurity scores, while larger herd sizes being associated with higher biosecurity scores (*p* ≤ 0.05). These findings suggest that biosecurity implementation remains limited across many farms, particularly among multi‐species and smaller‐scale operations, highlighting the need for targeted and context‐adapted biosecurity outreach and preparedness efforts in Puerto Rico, particularly in western regions located closer to ASF‐endemic areas in the Caribbean.

## 1. Introduction

The implementation of effective biosecurity practices is essential to protect swine production systems across North America from infectious transboundary threats, particularly high‐impact foreign animal diseases (FADs) such as African swine fever (ASF) [1]. This highly contagious disease represents one of the most critical FAD threats to the United States (US) swine industry, especially following the 2021 outbreaks in Haiti and the Dominican Republic, with some ASF outbreaks occurring ~140 km from the western coast of the US territory of Puerto Rico, which increases the risk of the disease spreading to the continental US [2]. The introduction of ASF in the US could result in severe economic consequences for the second‐largest world pork producer due to high mortality rates, large‐scale depopulation efforts, and trade restrictions [2]. Given the geographic proximity of recent outbreaks and the absence of an effective vaccine or treatment, prevention through enhanced biosecurity remains a key strategy to minimize the risk of ASF introduction and spread [3].

Planning and implementation of clearly structured, consistent, and widely adopted biosecurity plans that align with recognized standards are essential for mitigating the impact of a potential ASF introduction and enhancing disease preparedness [2]. The Secure Pork Supply [4] (SPS) Plan is a nationally recognized standardized biosecurity guideline developed for the US swine industry. This initiative, established through a public–private partnership involving the National Pork Board (Pork Checkoff), the U.S. Department of Agriculture Animal and Plant Health Inspection Service (USDA‐APHIS), and academia, provides swine producers with a framework to strengthen FAD readiness and continuity of business in the event of an outbreak [5]. The SPS Plan offers self‐assessment checklists and templates for developing site‐specific biosecurity plans, helping swine producers establish enhanced on‐farm biosecurity and maintain continuity of business during movement restrictions in case of an FAD introduction in the US [5]. However, the effectiveness of these tools depends not only on their availability but also on producers’ awareness about their existence and their willingness to adopt preventive attitudes and risk‐averse behaviors [1].

Adoption of on‐farm biosecurity practices remains largely voluntary and varies widely across farm types [6]. In farms for which gross cash farm income is ≥$350,000, including nonfamily farms and midsize or large‐scale family farms [7, 8], producers typically have greater resources to implement structured biosecurity protocols and maintain the necessary infrastructure [2]. In contrast, in residence and intermediate farms, which are classified as small family farms with <$350,000 in gross cash farm income [7], producers often face challenges such as limited financial resources, reduced access to training, lower awareness of recommended biosecurity measures, and lower motivation to use biosecurity due to lower perceived relevance [9]. Likewise, small‐scale producers tend to engage in higher‐risk practices such as trading at saleyards, utilizing food waste as feed, and undertaking minimal on‐farm biosecurity measures [10, 11]. These limitations can increase vulnerability to disease introduction and spread [2].

According to the US Department of Agriculture 2022 Census of Agriculture, most of the swine farms in Puerto Rico are family‐operated (86.08%) and typically occupy less than 50 acres [12]. It is estimated that ~36,069 hogs and pigs are distributed across 273 farms on the island. In 2021, Puerto Rico was designated as an “ASF Protection Zone” after the ASF outbreak in the Dominican Republic [13]. However, the adoption of biosecurity practices and the level of FAD preparedness among swine producers on the island remain understudied, highlighting the need to assess existing practices and identify gaps that could heighten the ASF introduction risk and compromise effective response.

The objectives of the study were to describe biosecurity practices across Puerto Rican swine farms using an adapted version of the SPS framework and to identify geographical areas of higher biosecurity‐related vulnerabilities on the island using a biosecurity scoring system. Furthermore, we aimed to evaluate the association between demographic predictors and biosecurity scores among the participating sites. The findings from the study will help identify priority areas for targeted biosecurity outreach and guide resource allocation for FAD preparedness, ultimately strengthening and protecting the swine industry in Puerto Rico.

## 2. Materials and Methods

### 2.1. Study Area and Population

Between September and December of 2024, swine producers in Puerto Rico were invited to voluntarily complete biosecurity preparedness forms through established contact lists maintained by three regional USDA Veterinary Medical Officers (VMOs). Prior to data collection, the VMOs received standardized training from the research team through three 1‐h virtual sessions on biosecurity plan creation and data recording to ensure consistency. Then, the VMOs reached out to producers in their assigned regions and scheduled a farm visit for in‐person data collection.

### 2.2. Data Collection

Data were collected using a standardized enhanced biosecurity plan adapted from the “SPS Biosecurity Plans” templates and translated into Spanish to reflect local production practices in Puerto Rico, including swill‐feeding systems (Supporting Information 1: File S1) [4]. The biosecurity preparedness plans captured information related to both current farm demographics and biosecurity practices as well as future planned improvements. The demographic section included data on the geographic location of the site, production type, herd size, and housing practices. The biosecurity practices section was organized around nine topics: (1) biosecurity manager, (2) training, (3) herd protection, including written plan, site access control, perimeter zones, and cleaning and disinfection, (4) vehicles and equipment, (5) personnel, (6) carcass disposal, (7) manure management, (8) rodent, fly, wildlife, and other animal control, and (9) feed management. Depending on site characteristics and applicability, each plan comprised ~60–70 items including a variety of question types, including open‐ended short answers, dichotomous (yes/no) questions, and multiple‐choice options to capture both quantifiable data and contextual insights and facilitate participation. The terminology used in the biosecurity plan are summarized in Table 1.

**Table 1:** Definitions of terms contained in the biosecurity preparedness plans.

Term	Definition
Perimeter buffer area (PBA)	Outer control boundary around the farm buildings to minimize the potential disease introduction near animals
PBA access point (PBAAP)	Specific, clearly marked entry/exit points for personnel, vehicles or equipment across the PBA
Line of separation (LOS)	Boundary between the animals and the exterior area (e.g., walls of a barn in an indoor or a fence in an outdoor system)
LOS access point (LOSAP)	Specific, clearly marked entry/exit points for personnel, vehicles or equipment across the LOS
Cleaning and disinfection (CD) station	Designated area, clearly marked to remove visible contamination and disinfect vehicles and equipment

Finally, the implementation of each component to date of questionnaire response was captured using a “Self‐Assessment Checklist,” which was adapted from the “SPS Plan: Enhanced Pork Production Biosecurity Checklist: Animals Raised Indoors” and “SPS Plan: Enhanced Pork Production Biosecurity Checklist: Animals Raised with Outdoor Access” [4] and likewise translated into Spanish (Supporting Information 2: File S2). The checklist consisted of 19 biosecurity components, each with three possible response categories: in place (all items are addressed in the biosecurity plan), in progress (some of the items are addressed in the biosecurity plan and are, or are capable of, being implemented), and not in place (the items have not been addressed in the biosecurity plan or are not capable of being implemented on the production site).

The producers completed both forms during an on‐site visit with the VMOs, recording information either digitally or on paper. The deidentified data were then shared with study investigators for analysis.

### 2.3. Data Management and Statistical Analysis

Data were digitized into Excel format using Python‐based optical character recognition [14], followed by manual verification to ensure accuracy. All data analysis and visualization were conducted in R software (Version 4.4.2) using the tidyverse suite of packages (including ggplot2 and dplyr), along with packages such as *sf* and *tigris* for spatial data processing [15].

For the biosecurity preparedness plans, a biosecurity score was computed using 37 variables representing key structural and operational biosecurity practices assessed across participating farms (Supporting Information 3: File S3). Variables not applicable to all farms, such as semen handling, were excluded from the scoring. Each variable was coded as binary, where the implementation of the practice was assigned a value of 1, and absence was assigned a value of 0. For scoring purposes, responses marked as “N/A” or left blank were coded as absence of the practice, acknowledging that this classification may include both true absence and nonresponse, which are both undesirable outcomes for the preparedness aspect of this work. The total biosecurity score for each farm was calculated as the sum of all variables, resulting in a maximum possible score of 37 points. To facilitate interpretation, total scores were standardized to a percentage scale (0%–100%) by dividing each farm score by the maximum possible score and multiplying by 100. Likewise, mean scores were calculated for each biosecurity practice across all surveyed farms and ranked in ascending order to identify the least and most implemented components. To visually evaluate geographical patterns in biosecurity implementation, farm‐level biosecurity scores were aggregated at regional levels according to the 2022 Census of Agriculture (2022 Puerto Rico Island and Regional Profiles) [12]. For each region, the mean biosecurity percentage was calculated as the average of farm‐level biosecurity percentages among all surveyed farms within the region. These values were then visualized using a map, applying a continuous color gradient scaled from the minimum to the maximum observed percentages to represent variation in biosecurity preparedness across regions.

For the “Self‐assessment Checklist,” each biosecurity item was classified in a response category (*in place*, *in progress*, *and not in place*). The number of farms per category was calculated for each item. Percentages were calculated using the number of nonmissing responses for each item as the denominator. Descriptive statistics were generated to summarize the implementation status of biosecurity practices.

In order to evaluate the association between farm characteristics and biosecurity scores, a multivariable linear mixed regression analysis was conducted in R software (Version 4.4.2) using packages including tidyverse, lme4, lmerTest, DHARMa, performance, ggeffects, marginaleffects, and broom [15], with biosecurity scores used as the outcome variable. Potential predictors considered during model selection included region, number of pigs in the region, backyard and commercial farm type, practice of swill feeding, housing type (indoor only, outdoor only, or mixed), presence of multiple species on the farm, and herd size. The region was used as a random effect to account for the clustering of farms within regions.

Prior to multivariable modeling, Spearman rank correlation analysis was conducted to assess pairwise associations among potential predictors and identify potential multicollinearity or redundancy. Correlations with an absolute Spearman’s *ρ* >0.80 were considered strong. Variables that were highly correlated or conceptually redundant were excluded before the regression analysis. Variance‐inflation factors were then examined among the retained predictors to further assess multicollinearity. The remaining predictors were evaluated individually using univariable mixed linear regression models. Predictors with a univariable association at *p* ≤ 0.20 were considered for inclusion in multivariable modeling. Multivariable model building was performed using a backward stepwise manner, and confounding variables were examined using a conceptual diagram as well as a change in coefficients of ≥ 20%.

Model results were reported as regression coefficients, 95% confidence intervals, and *p*‐values. Predictive marginal effects were estimated from the final mixed‐effects model to visualize adjusted predicted biosecurity scores by multiple‐species status and across the observed range of total pigs. Confidence intervals around the predicted values were presented to describe uncertainty in the marginal estimates.

## 3. Results

### 3.1. Study Population Characteristics

**Figure 1:**
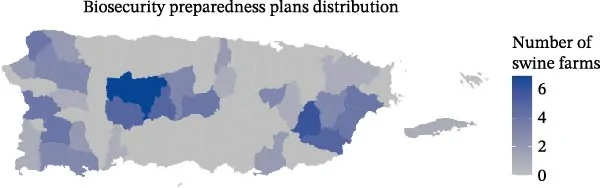
Map of the distribution of biosecurity preparedness plan respondents across municipalities in Puerto Rico. Each respondent represented a single farm included in the study.

Biosecurity preparedness plans were developed from 95 swine farms of 33 municipalities (out of 78 total) and 7 regions (out of 8 total) across Puerto Rico, mainly from the Caguas (*n* = 35), Utuado (*n* = 20), and Mayaguez (*n* = 15) regions (Figure 1).

Indoor housing systems were predominant among the surveyed sites, with 8.42% (*n* = 8/95) allowing outdoor access for pigs. The median (IQR) herd size was 32 (13–62.5), ranging from 1 to 665 pigs. The predominant production categories were grow‐finish pigs and gestating/lactating sows, each present in 73 of the 95 farms. Most farms were classified as backyard productions (77.89%, *n* = 74/95), and among these, 70.27% practiced swill feeding (*n* = 52/74). Approximately one‐third of the swine farms were multispecies (31.58%, *n* = 30/95), housing between two and four animal species, primarily cattle and poultry.

### 3.2. Biosecurity Preparedness Plans

All farms reported having a premise identification number (PIN) and a person responsible for the farm biosecurity or a biosecurity manager. However, only 6.32% (*n* = 6/95) of the farms indicated that the biosecurity manager gave instructions to the personnel before crossing the LOS. Approximately a third of the premises (35.79%, *n* = 34/95) indicated that workers received training on biosecurity, typically annually. Only one commercial farm reported having a site‐specific biosecurity plan. Notably, none of the farms reported having a specific veterinarian‐client relationship.

Approximately three‐quarters of farms reported having physical barriers restricting access to the operation (75.78%, *n* = 72/95). However, none of the farms reported the presence of entrance signs; two of them indicated plans to install signs in the local language. Likewise, almost all of them (98.94%, *n* = 94/95) use the same entrance for animals and people, suggesting a lack of animal‐specific loading chutes in the facilities. For pasture‐raised pigs, entry was mainly controlled using property fences; one farm indicated using zinc sheets to prevent contact with feral pigs, while another noted that pigs were kept free‐ranging with no physical barriers.

According to the plans, 55.78% of the farms (*n* = 53/95) reported a clearly delineated PBA limit. In agreement, almost half of the farms (46.31%, *n* = 44/95) reported that they implemented biosecurity measures to reduce contact with other animals, particularly wild pigs (88.63%, *n* = 39/44), pets (86.36%, *n* = 38/44), and other swine farms (84%, *n* = 37/44). A smaller proportion (36.36%, *n* = 16/44) indicated having biosecurity measures to prevent contact between poultry and pigs. However, among farms reporting multiple species, only 20% (*n* = 6/30) indicate having measures to minimize contact. Similarly, among farms allowing outdoor access, only one reported implementing biosecurity measures to prevent contact with other animals. Only 6.32% (*n* = 6/95) of the farms indicated keeping records of animal, vehicle, equipment, and personnel movements within the PBA, and 8.98% (*n* = 8/89) of them reported deliveries occurring inside the PBA. A large proportion of farms (90.52%, *n* = 86/95) reported the absence of and no plans to establish a CD station with the necessary supplies and equipment. Notably, only 5.26% (*n* = 5/95) of the farms indicated having a designated parking area clearly marked. A large proportion of farms (83.5%, *n* = 79/95) reported clearly marked access points to the LOS. However, approximately three‐quarters of them (77.21%, *n* = 61/79) indicated the absence of biosecurity practices when food, personal items, equipment, and supplies crossed the line.

For nonanimal transport vehicles and equipment, although most farms (95.79%, *n* = 91/95) reported using their own, 74.73% (*n* = 68/91) indicated that these were not cleaned and/or disinfected consistently prior to crossing the PBA. Likewise, 20% of farms (*n* = 19/95) reported allowing animal transport vehicles (e.g., livestock trucks and trailers) to cross the PBA; among these, 78.95% (*n* = 15/19) indicated that cleaning and disinfection were not performed. A small proportion of farms (3.16%, *n* = 3/95) reported garbage trucks entering the PBA/LOS weekly.

Notably, all farms reported having their own lawn care tools and other maintenance equipment. None of the farms reported sharing open feed containers with other farms, with most of them reporting using a closed container farm‐specific for feed management (83.16%, *n* = 79/95). Almost half of the participant farms, 48.42% (*n* = 46/95), used dedicated equipment for manure management or cleaned and disinfected equipment and vehicles from other sites. Most of the farms reported manure runoff, with only 23.15% (*n* = 22/95) using pits or oxidation ponds for manure management.

None of the farms reported the use of logbooks before crossing the LOSAP. Only 7.37% (*n* = 7/95) indicated the use of personal protective equipment (PPE) before entering the PBA. Similarly, only 9.47% (*n* = 9/95) noted the use of PPE before crossing the LOS. None of the farms reported shower‐in/shower‐out. Among the farms that indicated the use of PPE, 22.22% (*n* = 2/9) reported performing hand hygiene or removing PPE when exiting the LOS.

Most farms reported having boar presence (73.68%, *n* = 70/95); of them, 5.71% (4/70) reported using boars borrowed from a neighboring farm. Just one farm reported obtaining semen from farms with documented biosecurity practices. Moreover, almost half of farms (49.47%, *n* = 47/95) indicated a rodent control program. However, cats and snakes were reported on the site by 29.78% of them (*n* = 14/95), and less than 10% of farms (*n* = 9/95) noted an insect control program.

Finally, in case of an FAD outbreak, almost all farms (97.89%, *n* = 93/95) reported having a contingency plan to provide housing, feed, and veterinary equipment in case of interrupted animal movement. However, only about half of them (52.68%, *n* = 49/93) reported having the capacity to hold the entire herd during a 72‐h standstill (Table 2), and over one‐third (34.40%, *n* = 32/93) indicated that they would sustain totally or partially animals for only 1 day. Notably, only 5.26% (*n* = 5/95) could store manure for 72 h or more in the event of an outbreak (Table 2).

**Table 2:** Planned biosecurity preparedness practices for foreign animal disease (FAD) response on swine farms in Puerto Rico.

Biosecurity item (*n* = 95)	Frequency (% total)
Contingency plan^a^	**57 (60)**
≥ 3 days, all animals	49 (51.57)
≥ 3 days, partial	8 (8.42)
Carcass disposal	**62 (65.26)**
Government coordination	61 (64.21)
Inside PBA	1 (1.05)
Manure	**27 (28.41)**
Pits	21 (22.10)
Oxidation ponds	1 (1.05)
Storage ≥ 3 days	5 (5.26)
Feed truck	**23 (24.21)**
Inside PBA, cleaned and disinfected	14 (14.74)
Outside PBA	9 (9.47)

*Note:* Bold values indicate the main categories e.g. contingency plan 57 includes: ≥ 3 days, all animals (49) + ≥ 3 days, partial (8), and subsequently with the other bold categories.

^a^Respondents reported having a contingency plan in case of restriction of animal movement during 72 h in the event of an FAD outbreak.

In addition, only 14.13% (*n* = 13/92) of respondents noted having the necessary supplies for animal depopulation, while a substantial proportion of farms (64.21%, *n* = 61/95) indicated that carcass disposal would be coordinated with government authorities in the case of an emergency (Table 2), with burial most frequently identified as a potential method. None of the farms mentioned incineration, and just one farm mentioned composting. Additionally, 24% of farms (*n* = 23/95) indicated that feed delivery would either occur without crossing PBA or vehicles would be cleaned and disinfected before crossing in the event of an FAD outbreak.

### 3.3. Ranking of Biosecurity Practices

The mean biosecurity score amongst the whole study population was 42.8% (range: 26.3%–73.7%), with a median of 39.5% (IQR: 36.8%–47.4%). At the regional level, the highest mean biosecurity score was observed in Caguas (13 municipalities), followed by Utuado (4 municipalities) and Naranjito (2 municipalities). The lowest biosecurity score was observed in Arecibo (one municipality) and Mayaguez (five municipalities) (Table 3).

**Table 3:** Biosecurity ranking score by region in Puerto Rico.

Region	Total farms	Mean score (%)	Median score (%)	IQR (%)	Min. score (%)	Max. score (%)
Caguas	35	47.29	44.74	36.84–53.95	31.57	73.68
Utuado	20	44.47	44.74	42.11–45.39	34.21	55.26
Naranjito	5	43.16	42.11	39.47–44.74	34.21	55.26
Lares	4	40.13	36.84	36.84–40.13	36.84	50.00
San German	14	38.35	36.84	36.84–38.82	36.84	50.00
Mayaguez	15	36.14	36.84	32.89–36.84	31.58	47.37
Arecibo	2	31.58	31.58	28.95–34.21	26.32	36.84

**Figure 2:**
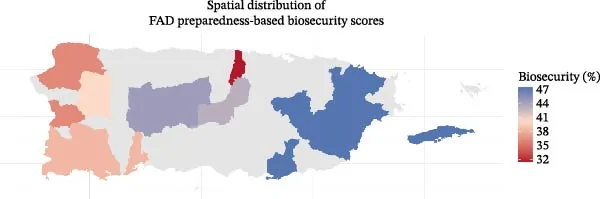
Map showing regional‐level biosecurity preparedness in Puerto Rico, with lower scores represented in red tones and higher scores in blue tones. Gray areas represent no available survey data. Regions were classified based on the mean biosecurity score proportion.

Higher‐scoring regions were concentrated in the eastern part of the island, while lower scores were observed mainly in the western and northern regions. Central regions showed intermediate values (Figure 2).

### 3.4. Biosecurity Implementation

**Figure 3:**
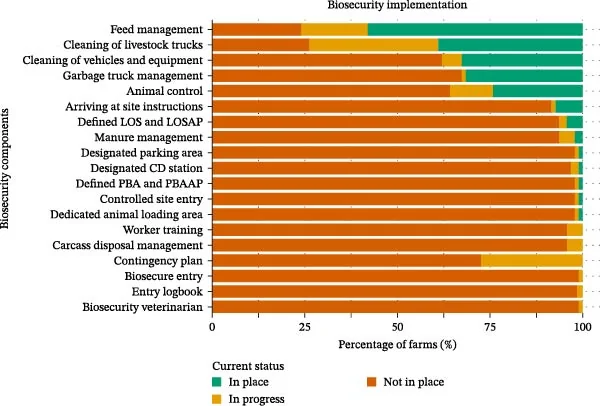
Implementation status of biosecurity plan components according to the “Self‐Assessment Checklist,” adapted from the SPS framework. Percentages were calculated based on nonmissing responses for each item. The number of responding farms was consistent across items (*n* = 95), except for the entry logbook item (*n* = 67). CD, cleaning and disinfection station; LOS, line of separation; LOSAP, LOS access point(s); PBA, perimeter buffer area; PBAAP, PBA access point(s).

Overall, a large proportion of farms reported that biosecurity practices were currently “not in place,” while only a small proportion indicated some or full implementation (Figure 3). Critical components such as veterinarian’s involvement in farm biosecurity, use of entry logbooks, implementation of biosecure entry procedures in the LOS, proper carcass disposal, and personnel training were almost entirely absent among the surveyed farms. Structural measures, including the establishment of PBA, LOS, CD stations, and designated parking areas, showed minimal implementation, with only a few farms reporting these practices as either “in place” or “in progress.”

In contrast, components related to routine operations, such as feed management (i.e., adequate feed hygiene, storage, and transportation using clean and disinfected vehicles), the cleaning and disinfection of both animal and nonanimal transport vehicles, and rodent, insect, and wildlife control, show comparatively higher levels of implementation or progress (Figure 3).

### 3.5. Statistical Model Results

Spearman rank correlation analysis identified strong correlations among several predictor variables. Mixed indoor–outdoor housing was highly correlated with indoor housing status (*ρ* = −0.94) and was therefore excluded from further modeling due to conceptual overlap. Commercial farm type was also excluded due to its moderate correlation with the backyard farm type (*ρ* = −0.53) and swill‐feeding status (*ρ* = −0.43). Based on the univariable screening criterion, backyard farm type, indoor housing, and total number of pigs in the region were not retained for multivariable modeling.

**Figure 4:**
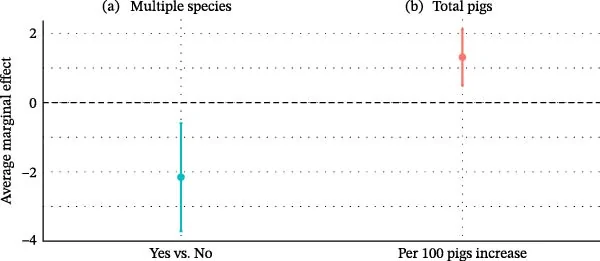
Average marginal effects from the final multivariable linear mixed‐effects model for biosecurity score (a) by multiple‐species status and (b) by herd size.

In the final linear mixed‐effects model, multiple‐species status and herd size were significantly associated with the biosecurity score, after accounting for clustering by region. Farms with multiple species were associated with a 2.15 points lower biosecurity score on average (95% CI: −3.735, −0.569; *p* = 0.008), holding the herd size constant and accounting for regional clustering. Additionally, for every 100 additional pigs on the farm, the biosecurity score increased by 1.3 points on average (95% CI: 0.5, 0.21; *p* = 0.002), after adjusting for multiple‐species status and accounting for region as a random effect (Figure 4).

## 4. Discussion

Biosecurity preparedness was assessed across swine farms in Puerto Rico to identify geographic areas with higher biosecurity‐related vulnerabilities, which could be associated with a higher risk of disease introduction and/or spread. Notably, most farms were found to have average biosecurity scores ≤ 50% using selected variables captured within our framework. Similar biosecurity levels have been found in small‐scale pig farms and family‐run operations from ASF‐endemic countries such as Cameroon, China, Tanzania, and Uganda, as well as in countries located near ASF‐affected regions, such as Slovenia, particularly among farms with outdoor access [16–20]. However, when compared with swine operations across the mainland US, gaps observed in Puerto Rico become even more evident. In contrast to a study conducted by Harlow [2], which evaluated 12,195 pig sites across 31 US states through the US Swine Health Improvement Plan (US SHIP), swine farms in Puerto Rico showed substantial deficiencies in farm entry biosecurity measures, particularly regarding visitor logbooks, entrance signs, and shower‐in/shower‐out protocols. For example, while only 1% of sites in the US SHIP study reported never requiring visitors to sign in, all farms evaluated in Puerto Rico lacked visitor sign‐in requirements and biosecure entrance signs. Likewise, none of the farms in Puerto Rico reported implementing shower‐in/shower‐out protocols, and only 7.37% reported using PPE. In comparison, 51% of sites across the mainland US required everyone to shower in, and 99.5% required changing clothes and footwear before entering the premises.

Interestingly, in our study, a discrepancy was observed between the absence of veterinarian involvement and the high proportion of farms reporting having a designated biosecurity manager. This suggests that although responsibility for biosecurity may be assigned, it is not necessarily supported by veterinary oversight or technical expertise. According to Gelalcha [9], veterinarians serve as trusted sources of information and play an important role in encouraging the adoption of biosecurity measures. In our study, the biosecurity plans were collected with the help of VMOs, suggesting a collaborative relationship between producers and animal health officials but not necessarily private practice veterinarians. Previous studies have also reported that the successful development and implementation of biosecurity protocols depend on establishing trustworthy relationships among all stakeholders and are essential for strengthening preparedness against emerging and FADs [21, 22]. The current lack of swine‐savvy veterinarians on the island may represent an additional challenge for the implementation and long‐term maintenance of effective biosecurity practices in Puerto Rico. This limitation highlights the need for additional training opportunities for small‐ and mixed‐animal practitioners, who are often perceived by farm staff as more trusted sources of information than external trainers [9], to strengthen their capacity to manage swine cases and implement on‐farm biosecurity protocols, particularly in the event of a disease outbreak.

Engaging producers proactively in biosecurity planning, training, and awareness initiatives through local networks tailored to the language and literacy level is critical to ensure that these measures are consistently applied and adapted to the unique conditions of each production system [9]. However, it is important to note that biosecurity implementation is influenced by socioeconomic and sociocultural challenges commonly faced by small‐scale production systems, including limited financial resources [21]. This needs to be taken into consideration, and financial incentives need to be allocated for successful disease introduction and spread prevention on the island.

In our study, although most of the farms reported some level of contingency capacity planning in the event of a FAD outbreak, the number of animals that could be housed, the duration for which they could be maintained under movement restrictions, and the capacity to store and manage manure appeared to be insufficient for effective outbreak management, especially in systems with limited infrastructure or reliance on runoff‐based practices for the latter. Likewise, although many farms indicated that depopulation and carcass disposal would be coordinated with government authorities, few reported having the necessary supplies, equipment, or preparedness measures in place to support on‐farm activities during an outbreak. It may also be the case that producers lack knowledge of acceptable methods related to those two needed practices. These findings are still in line with a previous study reporting high confidence among producers in their ability to contain an outbreak on their farms (even if that means relying on government officials to help), despite limited implementation of enhanced biosecurity practices [21]. This is an issue considering limited government resources, especially in cases where multiple cases of disease detection would be at play. Further training and development of ownership within producers regarding those types of activities are urgently needed in Puerto Rico.

In the US, enhanced biosecurity refers to a series of intensified disease prevention measures implemented to protect swine production from FAD and support the continuity of business during an outbreak [21]. In Puerto Rico, enhanced biosecurity measures and regulatory actions should have been strengthened following the establishment of the “ASF Protection zone” due to the island’s proximity to ASF outbreaks in the Caribbean [13]. Notably, western regions in our study exhibited lower biosecurity scores. This finding is particularly concerning, given the geographic proximity to ASF‐endemic areas in Haiti and the Dominican Republic and highlights the need for targeted outreach and preparedness efforts, particularly in the Mayaguez, Lares, and San German regions, as well as the northern region of Arecibo, which maintains a swine inventory of 10,000 pigs [12]. Likewise, the western region is composed predominantly of small‐scale backyard farms, with more than half of them housing multiple animal species. Small herd sizes and multispecies production systems were significantly associated with lower biosecurity scores in our analysis, suggesting that they may face greater challenges in planning and implementing effective biosecurity practices. Our findings agree with previous studies that have found farm size to be one of the most influential variables and a key determinant of biosecurity implementation [21], with smallholder pig farms having fewer biosecurity requirements compared to larger farms and a higher risk for ASF introduction and spreading among pig farms [22]. Furthermore, multi‐species sites may also pose a high risk of cross‐species disease transmission and exposure to new virus strains depending on the disease [23].

Puerto Rico is currently facing challenges associated with invasive populations of feral Vietnamese pot‐bellied pigs often found on properties with livestock operations across the island, which may further complicate swine disease prevention and biosecurity efforts [24]. In the context of ASF, interactions between domestic pigs and feral swine populations could increase opportunities for pathogen introduction and spread [25], particularly among farms with outdoor access, which represented 10% of the surveyed farms.

This project was not without limitations. First, because data collection relied on pre‐established connections, the study team was unable to personally gather the data. However, the training of the three VMOs together and the use of a standardized form likely reduced the potential for information bias. Second, selection bias may be present as participation in this study was voluntary in nature. As such, the population that did not participate in the study may systematically differ from the study participants in their biosecurity practices or preparedness level. The way this would affect the results is difficult to estimate, and the study findings should be interpreted and generalized with caution. Additionally, some producer‐reported practices, such as runoff channels (“entrecoleras”) and percolation trenches, were not considered adequate biosecurity measures within the scoring system since they can spread diseases if not properly designed [26]. Likewise, biological rodent control using predators, including cats and snakes, was not classified as an appropriate biosecurity measure due to the potential increased risk of pathogen introduction and interaction with other animal species [27]. Despite these limitations, an important strength of this study was the substantial farm participation achieved. Based on available estimates of swine premises in Puerto Rico, the study included nearly 35% of the estimated farm population of 273 premises [12], providing broad geographic representation and valuable insight into current biosecurity preparedness across the island.

## 5. Conclusions

This study provides a baseline understanding of the biosecurity practices in the swine industry in the US territory of Puerto Rico. Farms housing multiple species exhibited lower levels of biosecurity, while larger herd sizes were associated with higher biosecurity scores. Geographic variation in biosecurity implementation was observed across the island, with western areas showing potentially greater vulnerability to disease introduction. Although many producers reported some level of preparedness planning and a willingness to coordinate with government authorities during an outbreak, limited on‐farm resources, infrastructure, and specialized veterinary support may challenge effective disease response efforts. These findings emphasize the need for targeted, locally adapted training opportunities for local veterinarians and farm personnel to improve preparedness against FADs such as ASF in Puerto Rico.

## Funding

This project was funded by the USDA’s Animal and Plant Health Inspection Service through the National Animal Disease Preparedness and Response Program (Grant AP23VSSP0000C038).

## Ethics Statement

The study was deemed exempt by The Ohio State University Institutional Review Board under Category 4 (Secondary Research on Data or Specimens; Protocol Number 20252802). All analyses were conducted using deidentified data.

## Conflicts of Interest

The authors declare no conflicts of interest.

## Supporting Information

Additional supporting information can be found online in the Supporting Information section.

## Supporting information


**Supporting Information 1** File S1: English version of the enhanced biosecurity plan for the prevention of foreign animal diseases (FAD).

**Supporting Information 2** File S2: English version of the self‐assessment checklist: level of implementation of biosecurity plan components.

**Supporting Information 3** Variables included in the biosecurity score reported in the biosecurity plans.

## Data Availability

The data that support the findings of this study are available from the corresponding author upon reasonable request.
