# Supplementary material for: Factors Associated With Biosecurity Preparedness for Foreign Animal Diseases Among Swine Farms in the United States Territory of Puerto Rico
Source: Transbound Emerg Dis. 2026 Sep 14;2026:3769536. doi: 10.1155/tbed/3769536 (PMC13575490; doi:10.1155/tbed/3769536)
Supplement: Supplementary file 1 [file TBED-2026-3769536-s001.pdf]

File S2. Self-Assessment Checklist: Level of Implementation of Biosecurity Plan Components

For each section of the Biosecurity Plan, please indicate the level of implementation on your farm. Use the following definitions for each level of implementation:

In place: ALL items in the farm's biosecurity plan have been addressed.

In progress: SOME, but not all, items in the biosecurity plan have been addressed, or they can be implemented on the farm.

Not in place: The items have NOT been addressed, or they cannot be implemented on the farm.

Biosecurity Plan component	In place	In progress	Not in place
1. Contact information for the person responsible for biosecurity (veterinarian)	☐	☐	☐
2. Personnel training	☐	☐	☐
3. Farm entry is clearly marked	☐	☐	☐
4. The Perimeter Buffer Area (PBA) and PBA Access Points (PBAAP) are marked and clear	☐	☐	☐
5. The Cleaning and Disinfection (C&D) Station is clearly marked and equipped	☐	☐	☐
6. The designated parking area is marked and clear	☐	☐	☐
7. The Line of Separation (LOS) and its access point (LOSAP) is clear and marked	☐	☐	☐
8. Vehicles used to transport equipment, feed, etc. are clean and disinfected	☐	☐	☐
9. Vehicles used for animal transport are clean and disinfected	☐	☐	☐
10. The garbage collection vehicle does not cross the PBA/LOS	☐	☐	☐
11. Before crossing the LOS, personnel are informed about vehicle cleaning and personal hygiene	☐	☐	☐
12. An entry log is maintained for all people who cross the LOS	☐	☐	☐
13. Appropriate clothing and personal hygiene equipment are used before crossing the LOS	☐	☐	☐

Biosecurity Plan component	In place	In progress	Not in place
14. A contingency plan is in place for depopulation, housing, feed, and manage in the event of an outbreak	☐	☐	☐
15. Animals are loaded through an access point dedicated only to animals	☐	☐	☐
16. Carcass disposal plan	☐	☐	☐
17. In the event of an outbreak, there is a manure management plan	☐	☐	☐
18. Rodents, insects, wildlife, and other animals	☐	☐	☐
19. Feed (adequate hygiene, storage, and transport in a clean and disinfected vehicle)	☐	☐	☐
