# Supplementary material for: Factors Associated With Biosecurity Preparedness for Foreign Animal Diseases Among Swine Farms in the United States Territory of Puerto Rico
Source: Transbound Emerg Dis. 2026 Sep 14;2026:3769536. doi: 10.1155/tbed/3769536 (PMC13575490; doi:10.1155/tbed/3769536)
Supplement: Supplementary file 2 [file TBED-2026-3769536-s002.pdf]

File S3. Variables included in the biosecurity score reported in the biosecurity plans

ID	Variable	Definition
1	Biosecurity manager	A person responsible for biosecurity management was designated
2	Veterinarian	Veterinarian involvement was documented
3	Training	Workers were reported as having received training in biosecurity measures
4	BP Created	A biosecurity plan was documented as being in place
5	Access Barrier	Physical barriers at entrance points were mentioned in place
6	Entrance signals	Entrance signals were in place
7	PBA Limit	PBA was documented as being/will be clearly marked on the farm
8	PBAAP Limit	PBA Access Points were documented as clearly marked
9	Movement Register	All movements requiring entry into the PBA were documented as being/will be recorded
10	CD station	CD station was documented as being clearly marked
11	Parking Area	Designated parking area was documented as being/will be clearly marked
12	Animal Contact	Measures to minimize animal contact were documented for at least one animal species
13	LOS Limit	LOS was documented as being clearly marked
14	LOSAP Limit	LOSAP was documented as being/will be demarcated
15	LOSAP Biosecurity	Biosecurity measures were stated as being/will be taken when food, personal items, equipment and supplies cross the LOS
16	Vehicle Cleaning	All vehicles that do not contain live animals were reported as being/will be cleaned
17	Dedicated Vehicle	Vehicles are reported as dedicated to the farm
18	Animal Vehicle Cleaning	All empty animal transport vehicles were documented as being/will be cleaned and disinfected
19	Vehicle Outside PBA	Transport vehicles carrying live animals were stated as not crossing the PBA
20	Garbage Truck	Garbage trucks were stated as not crossing the PBA or as not coming to the operation
21	Dedicated Equipment	Equipment was reported as being dedicated to the farm
22	Visitors Instructions	Biosecurity measures were reported as being communicated to people crossing the LOS
23	Entry logbook	An entry log is reported as being available for people crossing the LOSAP
24	PPE PBA	At least one biosecurity measure was documented as being present at the PBA entrance
25	PPE LOS	At least one biosecurity measure was documented as being present at the LOS entrance
26	Depopulation	Animals requiring depopulation were documented as would be appropriately depopulated and disposed of on the operation

27	Depopulation Equipment	Equipment and supplies for depopulation were documented as being available
28	Contingency Plan	Water, feed and medical equipment were documented as being available
29	Contingency Time	Duration of availability of water, feed, and medical equipment was documented
30	Carcass Disposal	At least one proper carcass disposal method was reported
31	Manure Method	At least one manure storage method was reported
32	Manure Storage	Manure was documented as being able to be stored or managed in the event of an outbreak
33	Manure Equipment	Manure equipment was reported as being dedicated or cleaned and disinfected
34	Rodent Control	At least one proper rodent control method was documented
35	Insect Control	At least one insect control method was documented
36	Feed Storage	Feed was documented as being transported and stored in covered containers
37	Feed Truck	Feed trucks were stated as not crossing the PBA or cleaned and disinfected

* PBA (Perimeter Buffer Area), PBAAP (Perimeter Buffer Area Access Point(s)), LOS (Line of Separation), LOSAP (Line of Separation Access Point(s)), PPE (Personal Protective Equipment), CD (Cleaning and Disinfection), BP (Biosecurity Plan).
