# Supplementary material for: Factors Associated With Biosecurity Preparedness for Foreign Animal Diseases Among Swine Farms in the United States Territory of Puerto Rico
Source: Transbound Emerg Dis. 2026 Sep 14;2026:3769536. doi: 10.1155/tbed/3769536 (PMC13575490; doi:10.1155/tbed/3769536)
Supplement: Supplementary file 3 [file TBED-2026-3769536-s003.pdf]

File S1. Enhanced biosecurity plan for the prevention of Foreign Animal Diseases (FAD)

- National Premises Identification Number (Prem ID or PIN): ____

Coordinates
Lat:
Long:

- Premises Address: ____
- Farm type:
  ☐ Commercial ☐ Backyard ☐ Swill-feeding
- Number of pigs:
  Piglets ____ Weaned pigs ____ Finishing pigs ____ Boars ____
  Gestating/lactating sows ____ Total ____
- Pig housing type:
  ☐ Indoor ☐ Outdoor ☐ Both
- Other business operations on premises? If yes, what?

____

1. Biosecurity Manager and Written Plan

- Person responsible for biosecurity management on the farm (may be the owner).

Name

____

Phone ____ Email ____

- Veterinarian (name/company)

____

Phone ____ Email ____

2. Training

- Are workers trained on biosecurity measures, their importance, and the consequences of not following biosecurity protocols?
  ☐ Yes How often are they trained? ______________________________ ☐ No

3. Protecting the Pig Herd

- Does the farm have a biosecurity plan? [Yes] [No]
- The biosecurity plan is shared with other farms [Yes] [No]
  (If you answered Yes, please go to page 7 to complete the information for the other farms)

Site Entry

- Entrance points (including unused ones) are protected to restrict entry with a barrier type:

  ☐ Fence ☐ Gate ☐ Rope Other, which? ______

- If there are signs at these entrances, what do they say and in what language are they written?

  ______

- If there are pigs raised in pasture, the entrance to their space is a barrier of:

  ______

Perimeter Buffer area (PBA) and PBA Access Points (PBAAP)

- A perimeter buffer area (PBA) is an external control boundary of the farm to minimize the possible introduction of diseases near animal housing.
- The PBA is/will be marked on the farm with (e.g., fence and/or a rope between marked posts):

  ______

- Access to the PBA is restricted/will be restricted to ________ Access Point(s) (Number). The PBA Access Points are clearly marked with __________________________ (for example a rope) to restrict entries.
- All movements (animals, vehicles, equipment, people) requiring entry into the PBA are/will be recorded, and these records are kept/will be keep in______________________ (for example, an office).
- Deliveries are/will be made outside the PBA in (describe where) ___________________________________

Cleaning and Disinfection Station (CD)

- There is/will be an operational, clearly marked and equipped station located at ______________________
- The floor of the CD station is/will be __________________________ [DESCRIBE THE SURFACE – GRAVEL, PLASTIC COVERED]
- This site uses/will use _____________________ [DRAINAGE DITCHES OR OTHER PHYSICAL BARRIERS] to manage the runoff and ensure that the susceptible animals are not exposed
- The site has/will have access to all the equipment and supplies needed to operate the CD Station properly? [Yes] [No]
- The individuals listed below have received/will receive training on the proper selection and use of personal protective equipment, the principles of the CD to prevent the introduction of a disease, and can operate the CD Station effectively ____
- In the case of <u>inclement weather</u> (freezing temperatures, high winds) when the C&D Station cannot be operated, we have the following contingency plans ____ ____(Describe the alternatives)

Designated Parking Area

- The designated parking area is/will be clearly marked on the site with ________________________ [ROPES, SIGNS, ETC.]. It is located outside the PBA and away from animal areas.

Maximize Distance between Susceptible Pigs

- Steps are/will be taken to minimize nose-to-nose contact of pigs with:
  Pigs from neighboring farms? [Yes] [No]
  Backyard poultry (chickens, ducks, etc.)? [Yes] [No]
  Wild animals or pigs? [Yes] [No]
  Pets (dogs and/or cats)? [Yes] [No]

  Steps taken to minimize contact include/will include: ____

Line of Separation (LOS)

The Line of Separation (LOS) is a boundary between the animals and the external environment. The LOS can be in the form of walls in a closed housing system or a fence in an open or semi-closed housing system.
Select the options that apply:

- [__] Animals are raised indoors, so the walls of the building/wires make up the LOS.
- [__] Animals have outdoor access and the LOS is marked on the site with __________________________ (wires, ropes)

LOS Access Point

Select one of the options:

- [ ] The animals are kept enclosed, therefore the LOSAP is/will be demarcated with: [   ]
- [ ] The animals have outside access, therefore the LOSAP is/will be demarcated by a: [   ]
- Biosecurity measures that are/will be taken when food, personal items, equipment, and supplies cross the LOS include ____ (FOR EXAMPLE, CLEANING AND DISINFECTION, UV LIGHT, ETC.)
- Securing the buildings: ____________________________(name) is/are responsible for ensuring the buildings are secured and locked

4. Vehicles and Equipment

Non-animal vehicles (Equipment, feed delivery, etc.)

- All vehicles and equipment that do not contain live animals are/will be cleaned to remove the visible contamination and are/will be disinfected before crossing the PBA; otherwise, entry is prohibited? [Yes] [No]
- Are these vehicles and equipment shared with other farms? [Yes] [No]

Animal transport vehicles

- All empty animal transport vehicles are/will be cleaned and disinfected before arriving at the site (Outgoing animal loads) or before loading the animals for delivery to the site (Incoming animal loads). [Yes] [No]
- Is/will it be permitted for transport vehicles carrying live animals to cross the PBA without unloading the animals at the farm? [Yes] [No]

Other Equipment

The garbage truck:

- ☐ Cross the PBA/LOS. Indicate the frequency: ________________________
- ☐ Do not cross the PBA/LOS. Indicate the frequency: ________________________
- ☐ There is no garbage removal company, and the work is done by yourself.

- Is grass-cutting equipment and other maintenance equipment shared with other farms? [Yes] [No]

5. Personnel

Prior to arriving at the site

- Does/will the biosecurity manager ensure that all people required to cross the LOS receive instructions on vehicle cleaning (free of all manure/animal waste), personal hygiene (wear clean clothing and footwear after their last contact with susceptible animals), and information on the biosecurity measures they must follow once they arrive? [Yes] [No]

Entry logbook

- Is there an entry log for people crossing the LOS Access Points? [Yes] [No]
- Are entry times recorded for workers crossing the LOS access points? [Yes] [No]

Biosecure Entry/Exit Procedure

When entering the PBA, all individuals must/will:

- Wear disposable or disinfectable footwear [Yes] [No]
- Wear gloves or apply sanitizer [Yes] [No]
- No measures are in place

All individuals crossing the LOS must/will:

- Shower in and shower out [Yes] [No]
- Wear only site-specific coveralls and footwear in the LOS Access Points [Yes] [No]
- Wash hands, apply sanitizer, or wear disposable or disinfectable gloves [Yes] [No]
- Do the same procedure followed in reverse when crossing back over the LOS from the pig side, with site-specific clothing or coveralls and footwear left inside the LOS? [Yes] [No]
- No measures are in place

Reproduction on the farm:

- Semen arrives from farms with documented biosecurity practices that align with our biosecurity practices [Yes] [No]
- Semen arrives in packages that can be cleaned and disinfected, but the biosecure status is unknown [Yes] [No]
- Semen arrives in packages that are not clean or disinfected [Yes] [No]
- A boar from the same farm is used for breeding [Yes] [No]
- A boar from other farm is used for breeding [Yes] [No]

Contingency plan

In the event of an animal movement standstill lasting several weeks, this is our plan for maintaining biosecure animal management.

- Animals that need to be depopulated will be appropriately depopulated and disposed of on the operation [Yes] [No]
- Water, feed and medical equipment are available for ___________ (NUMBER) market pigs for a period of __________ (DAYS, WEEKS OR MONTHS) [Yes] [No]
- Housing and feed for the management of breeding-age pigs is enough for ___________ (NUMBER) pigs for a period of __________ (DAYS, WEEKS OR MONTHS) [Yes] [No]
- Equipment and supplies for depopulation are available if needed [Yes] [No]

Loading Animals

Select one of the following options:

- The animal loading/unloading area is NOT an entry point for people [Yes] [No]
- People enter each finishing barn through a main entrance that is also used for loading/unloading animals [Yes] [No]

6. Carcass Disposal

- In the event of an FMD outbreak, dead animals will be disposed of by the following method(s) (to prevent attracting wild animals, subject to local and/or federal regulations):

☐ Incineration ☐ Burial ☐ Composting ☐ Government authorities will be consulted

The location used/will be used for carcass disposal:

☐ Is/will be located outside the PBA ☐ Is/will be located inside the PBA
☐ There is/will be a truck available to transport the dead animals without entering the PBA
☐ There is/will be a truck available to transport the dead animals entering the PBA
☐ Government authorities will be consulted
☐ Other Explain ____

7. Manure Management

- *(Answer only for pigs raised indoors)* Manure is stored in:

☐ Pits ☐ Trenches ☐ Other Which one? ________________________________

- In the event of a disease outbreak, we can store the manure for ______ (DAYS, WEEKS, MONTHS). After this period, the method used to dispose of the manure is __________________ (METHOD).

Select one option:

- [__] Site-specific equipment is dedicated to manure within the PBA and is not shared with other sites.
- [__] All vehicles and equipment used for manure that come from other sites are cleaned and disinfected using heat or a chemical disinfectant and then dried.
- Animals are raised outdoors. Manure-handling equipment is not used [Yes] [No]

8. Rodents, Insects, Wildlife, and Other Pests

Insect and Rodent Control

The following measures will be implemented for insect and rodent control:

- This operation will use ________________ to prevent the movement of rats.
- Rodent traps are checked every (frequency) ________________ by ________________
- This operation uses ________________ to prevent the movement of insects, and _____________________ is responsible for implementing this insect-control plan.
- Grass and vegetation control is conducted every __________________

9. Feed

- To minimize contamination, feed for pigs will be transported and stored in:

☐ Bags ☐ A farm-specific covered container ☐ A covered container shared with other farms
☐ A covered wagon/trailer during transportation ☐ Other Specify (e.g., drones):___________________

To increase biosecurity during a disease outbreak, feed trucks and delivery equipment will be handled in the following manner:

- [__] The feed truck will make the delivery without crossing the PBA or LOS.
- [__] The feed truck will enter the PBA but will be cleaned and disinfected before crossing the PBA or LOS to deliver the feed.
- [__] None

Other farms' information in case of a shared biosecurity plan

Farm 2

- National Premises Identification Number (Prem ID or PIN): ☐
- Premises Address: ☐
- Farm type:
  ☐ Commercial ☐ Backyard ☐ Swill-feeding
- Number of pigs:
  Piglets ☐ Weaned pigs ☐ Finishing pigs ☐ Boars ☐
  Gestating/lactating sows ☐ Total ☐

Lat:

Long:

Farm 3

- National Premises Identification Number (Prem ID or PIN): ☐
- Premises Address: ☐
- Farm type:
  ☐ Commercial ☐ Backyard ☐ Swill-feeding
- Number of pigs:
  Piglets ☐ Weaned pigs ☐ Finishing pigs ☐ Boars ☐
  Gestating/lactating sows ☐ Total ☐

Lat:

Long:

Farm 4

- National Premises Identification Number (Prem ID or PIN): ☐
- Premises Address: ☐
- Farm type:
  ☐ Commercial ☐ Backyard ☐ Swill-feeding
- Number of pigs:
  Piglets ☐ Weaned pigs ☐ Finishing pigs ☐ Boars ☐
  Gestating/lactating sows ☐ Total ☐

Lat:

Long:
